# Maintenance of genome stability by Fanconi anemia proteins

**DOI:** 10.1186/s13578-016-0134-2

**Published:** 2017-02-22

**Authors:** Anna Palovcak, Wenjun Liu, Fenghua Yuan, Yanbin Zhang

**Affiliations:** Department of Biochemistry and Molecular Biology, University of Miami Miller School of Medicine, Gautier Building Room 311, 1011 NW 15th Street, Miami, FL 33136 USA

**Keywords:** DNA damage response, DNA repair, Fanconi anemia, FANCA, Genome instability

## Abstract

Persistent dysregulation of the DNA damage response and repair in cells causes genomic instability. The resulting genetic changes permit alterations in growth and proliferation observed in virtually all cancers. However, an unstable genome can serve as a double-edged sword by providing survival advantages in the ability to evade checkpoint signaling, but also creating vulnerabilities through dependency on alternative genomic maintenance factors. The Fanconi anemia pathway comprises an intricate network of DNA damage signaling and repair that are critical for protection against genomic instability. The importance of this pathway is underlined by the severity of the cancer predisposing syndrome Fanconi anemia which can be caused by biallelic mutations in any one of the 21 genes known thus far. This review delineates the roles of the Fanconi anemia pathway and the molecular actions of Fanconi anemia proteins in confronting replicative, oxidative, and mitotic stress.

## Genomic instability and Fanconi anemia

The study of genomic instability as a potent driver of malignancy has placed an ever-growing importance on understanding the molecular players that contribute to the protection of the genetic code within each cell. Genome instability is defined as an acquired state that allows for an increased rate of spontaneous genetic mutations throughout each replicative cell cycle [1]. Three different types of genomic instability are recognized: (1) microsatellite instability (MI) which is characterized by random insertions or deletions of several base pairs in microsatellite sequences. MI is commonly observed in hereditary colorectal carcinomas, with defects in mismatch repair proteins. (2) Nucleotide instability causes subtle sequence changes as a result of DNA polymerase infidelity, aberrant base excision repair (BER) or nucleotide excision repair (NER). (3) Chromosomal instability (CIN) is the most frequently observed type of genome instability and has the greatest potential to lead to oncogenic transformation. CIN is responsible for translocations, inversions, deletions, aneuploidy, and other chromosomal changes that can vary from cell to cell [1]. The significance of these genomic instabilities in promoting pro-oncogenic events is highlighted by the presence of at least one type in almost all cancers at every stage of progression, and in hereditary and sporadic cancers alike [2]. The ubiquity of genomic instability in tumor cells has called for its inclusion as a hallmark of cancer, although the mechanism by which it arises has shown to differ between cancers of genetic or spontaneous origin. Germline mutations of DNA damage repair genes predispose individuals to cancer development through acquisition of a “mutator phenotype”. A mutator phenotype allows for higher rates of genetic mutation to occur due to reduced or absent expression of ‘caretaker genes’ that function in ensuring that aberrant DNA sequence changes are corrected before being passed on to newly divided daughter cells. An accumulated amount of unrepaired damage and errors could then result in the ability to avoid checkpoint mechanisms and further mutate genes that are essential for regulating cellular growth signaling and proliferation. The origin of sporadic cancers is much more elusive, but is hypothesized to arise from replication stress and its related mechanisms [3]. Because little is known about the mechanisms of sporadic oncogenesis, hereditary cancer-predisposing diseases serve as excellent models for studying the proteins and pathways that are altered to be tumorigenic.

Fanconi anemia (FA) is one such disease model that holds the potential to uncover the activities of a group of proteins that have prominent roles in genome maintenance. FA is a rare, inherited chromosomal instability disorder caused by biallelic mutation in one of the 21 known complementation groups [4–9]. Because FA proteins mediate DNA interstrand crosslink repair, cells from affected patients show hypersensitivity to crosslinking agents such as Mitomycin C (MMC), Diepoxybutane (DEB) and Cyclophosphamide. The increased amount of chromosome breaks observed in FA cells upon treatment with DEB is used as a diagnostic tool to confirm that an individual does indeed harbor a mutation within one of the Fanconi anemia genes [10]. Consistent with the association of genome integrity with carcinogenesis, FA patients suffer from myeloid leukemias, liver tumors, head and neck carcinomas, and gynecologic malignancies more frequently and at a younger age than the general population [11, 12]. Blood related pathologies contribute to the most severe symptoms of FA as the probability of developing myelodysplasia and acute myeloid leukemia (AML) in FA patients is 30–40% by 40 years of age. Sequencing studies and FISH analysis have shown that amplifications of certain oncogenes due to chromosomal translocations are responsible for blood cancers in FA patients [13]. It was found that hematopoietic regulating transcription factor *RUNX1* is often altered as a result of balanced and unbalanced translocations in both FA and non-FA cases of AML, indicating that the etiologies of FA-associated genome instability are relevant for studying carcinogenesis in populations unaffected by FA [13]. The functions of the Fanconi anemia proteins can be classified into several separate groups based on each one’s role in their canonical pathway of interstrand crosslink repair. Group 1 is classified as the core complex, which consists of FANCA, FANCB, FANCC, FANCE, FANCF, FANCG, FANCL, FANCM, along with Fanconi Anemia Associated Proteins FAAP100, FAAP20, FAAP24 [5, 14]. Although the entire function of the core complex is not completely understood, multimerization of the Group 1 proteins is necessary for monoubiquitination of FANCD2–FANCI upon recognition of cross-linked DNA in the presence of an ubiquitin conjugating enzyme UBE2T/FANCT [15–20]. The group 2 FANCD2–FANCI or the ID complex, once activated by monoubiquitination, recruits group 3 DNA repair factors that are critical for resolving interstrand crosslinks sensed during S phase [21]. Group 3 proteins are the downstream repair factors DNA endonuclease XPF/FANCQ, nuclease scaffolding protein SLX4/FANCP, translesion synthesis factor REV7/FANCV, and Homologous Recombination Proteins BRCA2/FANCD1, BRIP1/FANCJ, PALB2/FANCN, RAD51C/FANCO, RAD51/FANCR, BRCA1/FANCS, and XRCC2/FANCU [7, 22–24] (Biallelic mutations of XRCC2 are only found from cells derived from a previously identified patient, thus more XRCC2 patients are needed to confirm XRCC2 as a FA gene). The repair capacities of FA proteins in the occurrence of interstrand crosslinks, in themselves, contribute to the proteins roles as ‘caretakers’ and keepers of genome stability. However, recently elucidated functions of these proteins in other pathways broaden the spectrum of ways that they contribute to genome stability as well as ways that they may contribute to the mechanisms of sporadic cancers.

## FA proteins function in overcoming replication stress

Replication stress occurs when a structure or lesion present within DNA obstructs replication machinery and causes stalling [25]. The source of replication stress must be repaired without alterations to the genomic sequence in a timely manner in order to avoid deleterious fork collapse. Fork collapse increases the chances of producing a genetically unstable cell by allowing for incomplete replication and subsequent deletions and translocations that perpetuate these replication errors throughout remaining cell divisions.

### Interstrand crosslink repair

**Fig. 1:**
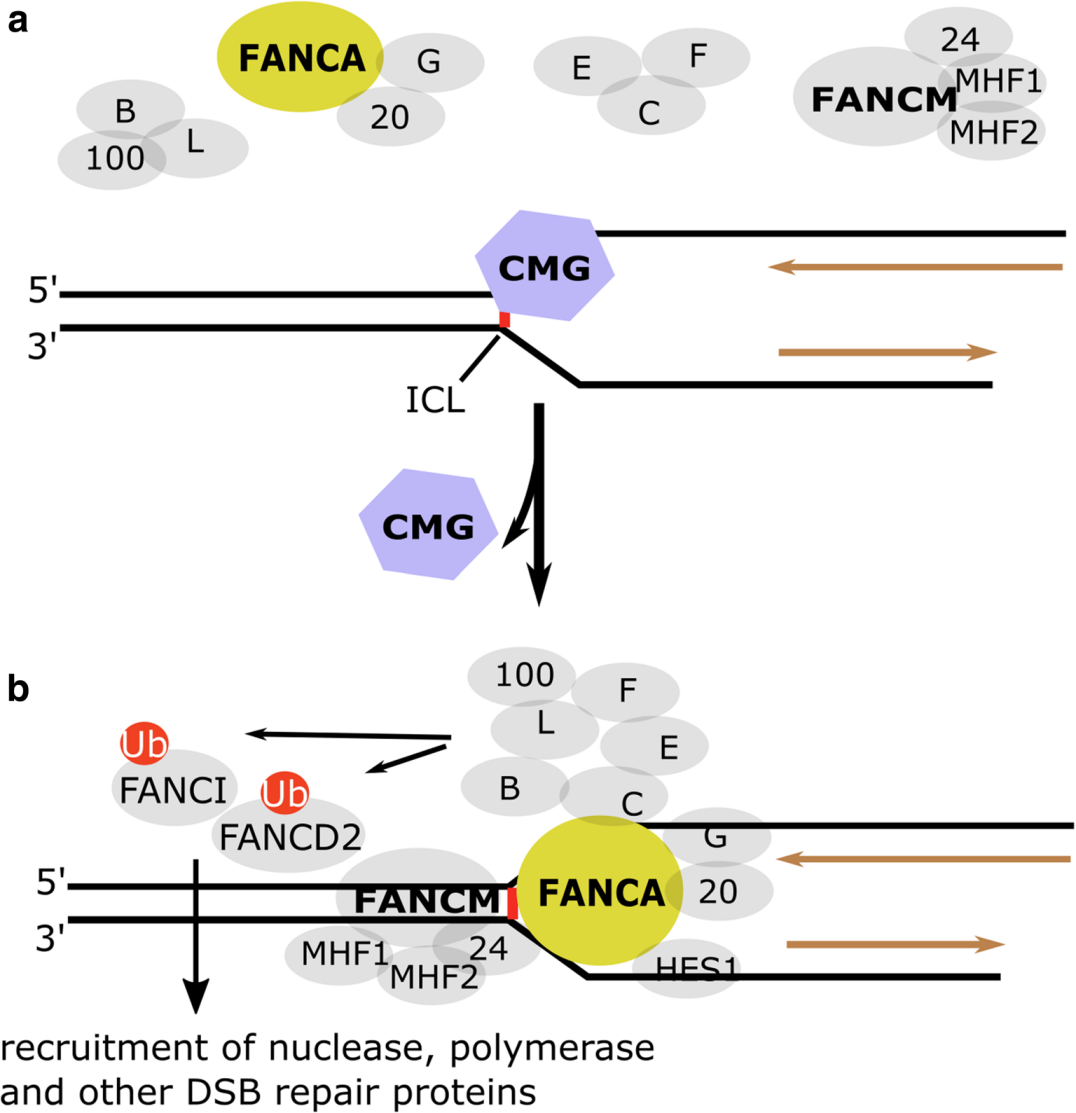
Interstrand crosslink sensing by the Fanconi anemia pathway. **a** The CMG helicase encounters ICL damage at the replication fork. **b** FANCM could be the primary factor in recognizing the interstrand crosslink upon replication folk stall. After damage verification presumably by FANCA, assembly of the FA core complex on the ICL site provokes the ubiquitin ligase activity of FANCL and results in monoubiquitination of FANCD2–FANCI complex, which further recruits downstream nucleases, polymerases, and DSB repair factors for the procession and repair of ICL

One of the primary protective roles of FA proteins is their assistance of replication fork recovery at stalled interstrand crosslinks (ICLs). ICLs completely block replication fork progression by covalently linking both strands of the DNA double helix, creating a lesion so cytotoxic that a single cell can withstand only 20–60 at one time [26]. Exogenous sources of ICLs include chemotherapeutic agents Mitomycin C, Diepoxybutane, and Nitrogen Mustards. ICLs can also form endogenously through linkage of the C4′-oxidized abasic site (C4-AP) with an adenine (dA) site present at the position opposite the 3′ neighboring nucleotide [27, 28]. It has also been demonstrated in vitro that aldehydes are able to react with the exocyclic amino group of a DNA base, forming an aldehyde/DNA adduct that can further be processed into an ICL [29, 30]. There are abundant sources of endogenous aldehydes such as acetaldehyde produced from ethanol metabolism or malondialdehyde, and crotonaldehyde from lipid peroxidation [30]. In vivo studies have shown bone marrow cells of FANCD2 null mice to be hypersensitive to aldehyde accumulation, which supports the necessity of ICL repair by the FA pathway for management of the damage caused by these reactive endogenous species [31]. The first event of ICL repair occurs during S phase and requires convergence of two replication forks on an interstrand crosslink [32]. When the replication machinery stalls at an ICL, the CMG helicase complex is unloaded from chromatin in a BRCA1 (FANCS)-BARD1 dependent manner [33] (Fig. 1). It is proposed that FANCM is responsible for recognizing the ICL lesion, and then inducing the recruitment of the downstream factors within the FA pathway that are necessary to carry out repair [34], the events of which take place through the following mechanism: FANCA, FANCG, and FAAP20 associate to form one subcomplex within the FA core, while FANCE, FANCF, and FANCC form another subcomplex [35] (Fig. 1a). The exact purpose of this subcomplex formation is unknown, however the multimerization of 8 FA proteins (FANCA, FANCB, FANCC, FANCE, FANCF, FANCG, FANCL, FANCM) along with 5 FA-associated proteins (FAAP100, FAAP24, HES1, MHF1, and MHF2) results in a 13-subunit ubiquitin ligase that functions to monoubiquitinate the FANCD2–FANCI heterodimer [34, 36] (Fig. 1b). Although recent in vitro studies have suggested that removal of one of the subcomplexes (A-G-20 or F-E-C) weakens the ubiquitination of the FANCD2–FANCI complex, removal of both subcomplexes is necessary to completely ablate the ubiquitin ligase activity of the core complex [35]. Because FANCA has DNA binding activity and regulates MUS81–EME1 endonuclease activity in an ICL damage-dependent manner [37, 38], it could contribute to chromatin localization, ICL damage verification, and the attachment of the subcomplex to DNA at the site of lesion. The ubiquitin ligase function of FANCL is dependent on its catalytic subcomplex consisting of FANCB and FAAP100 (B-L-100), which are also present within the multi-subunit core (Fig. 1b). The mechanism that explains the ability of these proteins to provide the catalytic activity of the B-L-100 subcomplex is unknown at this time [35], but earlier work has shown that FANCL and FANCB are required for the nuclear localization of FANCA, suggesting that at least one role of the catalytic core subunit functions to ensure proper assembly of the entire FA core [39]. The A-G-20 and B-L-100 subcomplexes form around FANCM once localized to the nucleus where they are both stabilized by FANCF, allowing for the formation of the entire core complex that is able to direct FANCL to FANCD2–FANCI for monoubiquitination [39]. The phosphorylation of FANCA on Serine 1449 in a DNA-damage inducible manner is dependent on ATR and has also been shown to promote FANCD2–FANCI monoubiquitination and downstream FA pathway function through a mechanism yet to be elucidated [40].

Ubiquitinated FANCD2–FANCI is required for its own recruitment to the ICL site, as well as for the promotion of the nucleolytic incision flanking the crosslink [22]. The exact components and mechanism surrounding the endonucleolytic cleavage of an ICL is not yet clear, however it has been shown that XPF–ERCC1, MUS81–EME1, FAN1, and/or SNM1 are necessary for ICL incision, which helps to facilitate unhooking of the structure [26, 38, 41–53]. It has also been recently shown that the SLX4 scaffolding protein forms a complex with XPF–ERCC1 to stimulate its fork unhooking activity [54]. An unidentified translesion polymerase inserts a base opposite the unhooked lesion in order for bypass to occur on the leading strand [26]. MUS81–EME1 then processes the stalled replication fork on the lagging strand into a double stranded break, serving as a programmed intermediate [43]. The leading strand is then extended by the Rev1–pol ζ complex [55] and ligated to the first downstream Okazaki fragment which further functions as a template for repair of the double stranded break, incurred on the lagging strand, through homologous recombination [56]. In the case of proper ICL repair by the FA pathway, the lesion is repaired in a timely manner while maintaining the fidelity of the genetic code where it had originally interfered. In the absence of one of the key components of the FA mediated pathway of ICL repair, aberrant end joining results in radial chromosome formation that is characteristic of Fanconi anemia cells [34, 57].

### Repair pathway choice

There is evidence to show that the FA pathway may have a role in preventing chromosomal instability by determining the repair pathway choice that occurs at the DSB generated during ICL repair. Inappropriate nonhomologous end joining (NHEJ) results in the ligation of free DNA ends that could originate from differing locations, making it responsible for the translocations observed in FA deficient cells. Interestingly, knockout of factors necessary for NHEJ alleviates much of the interstrand crosslink sensitivity observed in FA cells, demonstrating that one of the critical roles of Fanconi anemia proteins is the suppression of aberrant end joining that leads to chromosomal instability [58]. It has been reported that Ub-FANCD2 promotes HR and represses NHEJ by localizing histone acetylase TIP60 to the damaged chromatin, which then acetylates H4K16 and effectively blocks binding of 53BP1 to the neighboring dimethylated histone H4K20 (H4K20Me2) [59]. 53BP1 association with H4K20Me2 blocks end resection, the initiating event of HR, allowing NHEJ to proceed as the method of repair [59]. Ub-FANCD2 is required for impeding the ability of 53BP1 to promote NHEJ so that HR can faithfully restore the damaged genomic sequence. Additionally, the resection-promoting protein CtIP has been shown to interact with monoubiquitinated FANCD2. This interaction allows for end resection of the exposed strands during double stranded breaks, which is the committal step in promoting a homology directed repair pathway over error-prone end joining. The ability for Ub-FANCD2 to mediate CtIP end resection shows that the FA pathway is required for initiating the faithful repair at a double stranded DNA break [60].

### Promotion of replication fork stability

Fanconi anemia deficient cells have an impaired ability to restart replication at collapsed forks resulting from encounters with crosslinking lesions and DSBs [61]. Additionally, depletion of FANCA or FANCD2 causes DSB accumulation during normal replication, indicative of prolonged replication fork stalling [62]. Although evidence existed to support the ability of the FA pathway to stabilize replication forks, it was not until recently that the elucidation of its interaction with FAN1 began to provide an explanation for how FA proteins accomplish this mechanistically. It has now been discovered that replication fork stability is achieved through the recruitment of FAN1 to stalled forks in an Ub-FANCD2 dependent manner [63]. FAN1 has been shown to interact with FANCD2 through its N-terminal UBZ binding domain, and has structure specific exonuclease activity with 5′ flaps as a preferred substrate [64]. Mutations in *FAN1* are associated with ICL sensitivity and chromosome instability. However, the disease in FAN1-mutated individuals present as Karyomegalic Insterstitial Nephritis rather than Fanconi anemia. This differing phenotypic manifestation could indicate that FAN1 may have a secondary role in resolving ICLs, but its primary function is not limited to this [64, 65]. Consistent with this explanation, the recruitment of FAN1 by Ub-FANCD2 has been shown to be necessary for protecting stalled replication forks even in the absence of ICLs, although the mechanism of action for this protective ability is unknown. Also, FAN1 is not required for ICL repair, but still collaborates with FANCD2 to prevent replication forks from progressing when stalled at sites of DNA damage [63], a function that is required for preventing chromosomal instability. The abilities of the FA pathway in remediating replication dysfunction through recruitment of repair proteins, such as FAN1, underline its essential role in preventing aberrant processing of DNA lesions encountered by the replication machinery.

### Fanconi anemia pathway and Bloom helicase

Another interesting FA-mediated mechanism of genome maintenance involves the interaction of Ub-FANCD2 and Bloom helicase (BLM) and their co-localization to the nucleus when replication forks stall. BLM is mutated in Bloom syndrome, an inherited genomic instability disorder similar to Fanconi anemia in its childhood cancer predisposition as well as the presence of aberrant chromosome structures [66]. Earlier work has shown that a BLM complex, consisting of BLM, RMI1, RMI2, and TopoIIIα, associates with 5 of the FA (-A, -C, -E, -F, -G) proteins to form an even larger complex termed BRAFT, which displays helicase activity dependent on BLM [67]. Later it was shown that the association of the BLM complex with FA core proteins (FANCA, FANCE, FANCF) is mediated by a mutual interaction with FANCM where FANCM acts as a link between the two complexes [68]. This protein–protein interaction between FANCM and the BLM/FA complexes is required for resistance to MMC sensitivity as well as for foci formation at stalled replication forks [68]. Most recently it has been discovered that motif VI of BLM’s RecQ helicase domain contributes to regulation of the activation of FANCD2. Evidence for this was shown in U2OS cells with BLM knocked down via shRNA and then transfected with an expression plasmid containing mutations in motif VI that have also been documented to occur in certain cases of human cancer. Results from this transfection showed that deletions and point mutations within region Y974Q975 of BLM motif VI caused FANCD2 activation to be compromised after UVB treatment. Additionally, a proliferation assay showed reduced survival in mutant motif VI-transfected U2OS cells upon UVB and MMC treatment [69]. Together, these separate studies corroborate a collaborative effort for BLM and FA pathways in response to replication stress, although the exact function carried out through this interaction in replication-associated repair seems to remain largely a mystery. It appears that BLM is responsible for elevated sister chromatid exchange (SCE) independently of the FA pathway, but BLM does assist FA proteins in ICL repair [70]. BLM has shown the ability to resolve holiday junction structures during HR, and FA proteins have demonstrated their own roles in facilitating HR [71], possibly indicating that the functional interaction between these two complexes relates to maintenance of HR events that take place at the DSB that is produced during ICL removal. There are many missing pieces to the puzzle of the relationship between the BLM and FA pathways; more research is needed to fully detail the events that characterize BRAFT and the conditions that require BLM and FA proteins to work together.

### Coordination of the alternative end-joining pathway of repair

A study has confirmed a role of the FA pathway in supporting the Alt-EJ method of repair in cancers with BRCA1 or BRCA2 deficiencies. Alt-EJ is not a commonly utilized repair pathway in normal cells, but is thought to be responsible for translocations resulting in severe genomic instability observed frequently in cancer. Alt-EJ has been proposed as a culprit for these genomic rearrangements due to the sequences of microhomology that are present at chromosomal break-point fusion sites that are also characteristic of the microhomology sequences thought to mediate the ligation step in the microhomology mediated end joining (MMEJ) subtype of Alt-EJ [72]. Alt-EJ is proposed as an alternative to C-NHEJ making it primarily active during G1, although it can serve as an alternative repair mechanism to homologous recombination in S phase as well [72]. While the reasons that the extremely deleterious Alt-EJ undertakes repair of DSB in the place of HR or NHEJ is still heavily debated, it has been proposed to arise as a backup mechanism that takes place in cases when other pathways, such as HR and NHEJ, cannot be carried out [73]. BRCA1/2 cancers have been shown to rely on Alt-EJ for stabilization of replication forks and DSB repair in the absence of functional HR. The promotion of Alt-EJ in place of HR allows for survival of these cancers when faced with cytotoxic DNA damage and replicative stress perpetuated by a genomic instability phenotype. Examination of FANCD2 during DNA repair events in BRCA1/2 tumors has revealed its ability to recruit Pol θ and CtIP, factors that are critical for the Alt-EJ pathway. Monoubiquitination of FANCD2 was shown to be required for its coordination of these essential Alt-EJ components. FANCD2 also stabilizes stalled replication forks in BRCA1/2 deficient cancers, permitting their viability in extremely unstable genetic conditions [74]. Not only does this discovery establish a role for FANCD2 in promoting the error-prone Alt-EJ pathway, but also reveals the possibility of the FA pathway proteins serving as potent therapeutic targets in HR-defective malignancies.

### R-loop resolution

Another example of FA canonical function involves the resolution of replication forks that are blocked by transcription intermediates such as R-loops. R-loops are extremely stable, 3-stranded RNA:DNA hybrids generated by RNA Polymerase during transcription and serve as a source of genomic instability. They have physiological relevance in cellular processes such as class-switch recombination and mitochondrial DNA replication, but are also rare transcription events capable of causing altered gene expression and replication fork stalling when they encounter the replication machinery [75, 76]. Although the exact mechanism of R-loop induced genomic instability is not entirely known, they may induce harmful chromatin condensation capable of erroneously silencing gene expression [77]. Their elimination is necessary for maintaining faithful replication by preventing collision with replication machinery in addition to preventing faulty heterochromatin formation. Evidence for the FA pathway’s ability to facilitate R-loop removal is seen by the persistent R-loop accumulation in FANCD2 and FANCA depleted cells [78]. RNA:DNA hybrids are known substrates for RNase H1 and treatment of FANCA^−/−^ lymphoblast patient cell lines with RNase H1 reduces FANCD2 nuclear foci accumulation [78]. Another study has shown that FANCD2 monoubiquitination and foci formation was significantly reduced upon treatment with a transcription inhibitor. This supports the idea that a transcription intermediate, likely an R-loop, is responsible for activating the FA pathway to participate in repair [79]. Although the monoubiquitination of FANCD2 does indicate that the canonical FA pathway is involved in R-loop removal, the role of how this pathway regulates R-loop accumulation is not completely clear. The exact proteins that fulfill many aspects of this process remain to be identified, but the individual properties of some FA proteins would make them excellent candidate genes. Recognition of the R-loop structure, for example, could be carried out by FANCA, which has been shown to have RNA binding activity [37].

## Role of FANCA in maintaining genomic stability

Mutations in any of the 21 complementation groups cause an affected individual to present the standard phenotypes associated with Fanconi anemia. However, FANCA is found to be responsible for approximately 64% of FA cases [80–83] which raises great curiosity about the potential significance this protein may hold in maintenance of genome integrity. As seen in patients carrying mutant FANCA, even different patient mutations within the same protein can have varying phenotypes. FANCA patient studies revealed that a monoallelic delE12–31 mutation was associated with higher rates of AML or MDS as well as anatomic malformations not observed in other FANCA mutations [84]. Some patient-derived FANCA mutants still show the ability to monoubiquitinate FANCD2, albeit at lower levels, yet still display characteristic FA phenotypes and disease progression [85]. FANCA is emerging as a more interesting protein than previously evaluated due to its recently elucidated biochemical properties that are implicated in overcoming multiple forms of replication stress, as well as promoting different pathways of DNA repair.

**Fig. 2:**
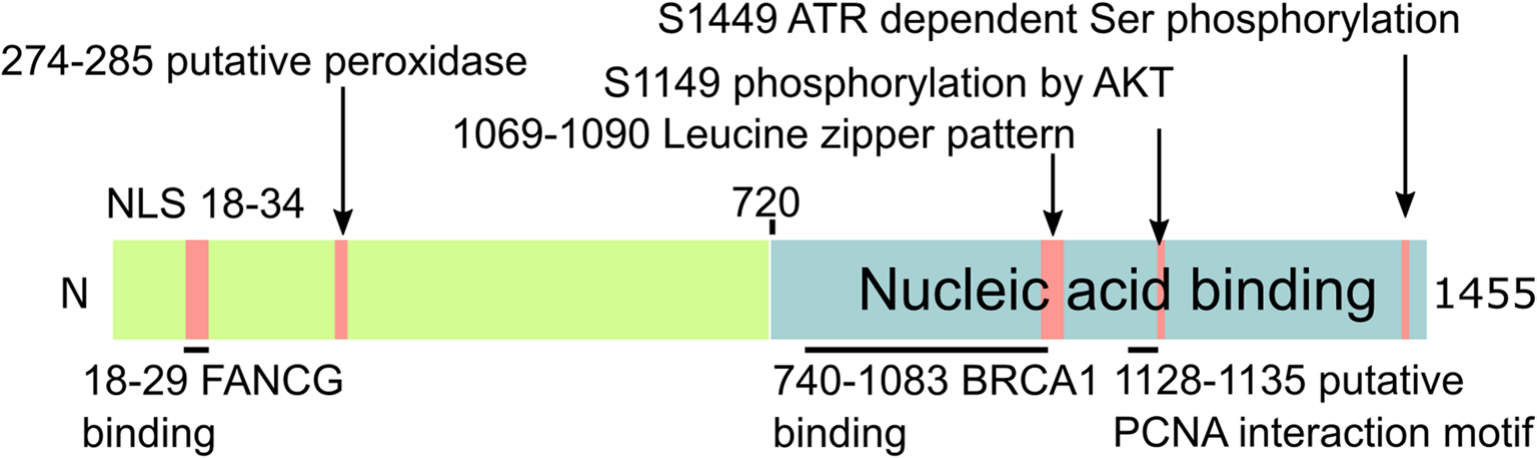
Structure and functional annotation of FANCA (NP-000126). The intrinsic nucleic acid binding activity resides in the C-terminal domain 720–1455. The N terminus contains the nuclear localization signal (18–34 or 19–35) [164] and was found crucial for both FANCG and FANCC interactions. The region 740–1083 mediates the interaction with BRCA1. Other putative functional remarks include a peroxidase (274–285), a PCNA interaction (1128–1135) motif, and a partial leucine zipper (1069–1090). Proteomic evaluation reveals multiple phosphor serine on FANCA, among which S1149 and S1449 were characterized as AKT and ATR substrates and critical for FANCA functions

FANCA contains 1455aa with a molecular weight of 163 kDa. It has a leucine zipper-like motif between amino acids 1069 and 1090 [86] and a bipartite Nuclear Localization Signal in its N-term that is activated by direct binding with FANCG [87] (Fig. 2). Disease-causing mutations are mostly found in the C-terminal, which has been shown to be required for the DNA binding function of FANCA [37]. While much still remains to be discovered about the biochemical properties of FANCA, recent research has uncovered some very interesting functions of this protein separate from its role in the canonical FA pathway. Due to its increasing importance in genome preservation, the following section will specially focus on the roles of FA proteins in maintaining genomic stability through absolving replicative, oxidative, and mitotic stress.

### Regulations of MUS81–EME1 endonuclease activity by FANCA

Our lab has shown the ability of FANCA to mediate the incision step of ICL repair by regulating MUS81–EME1 in vitro [38]. MUS81–EME1 is a structure specific heterodimeric endonuclease complex with substrate preference for 3′ flap structures with a 5′ end 4 nucleotides away from the flap junction [88]. We have also demonstrated that MUS81–EME1 was able to cleave the 5′ leading strand at the site of an ICL, 4–5 nucleotides away from the junction site [38]. FANCA regulates cleavage activity of MUS81–EME1 by recruiting the heterodimer when a verified ICL is present at the site of replication fork stalling, or FANCA will inhibit MUS81–EME1 accumulation in the case of non-ICL damage [38]. FANCA protects the genome in this manner by preventing MUS81–EME1 from creating unnecessary double strand breaks. Interestingly, a different in vivo study showed increased cases of embryonic lethality in FANCC/MUS81 double knockout mice. FancC^(−/−)^/Mus81^(−/−)^ mice also displayed developmental abnormalities, such as craniofacial malformations and ocular defects, that mimic human FA patient phenotypes and are not recapitulated in mouse disease models carrying FA mutations alone [89]. This could suggest that other FA proteins, in addition to FANCA, participate in the regulation of MUS81–EME1 in its roles of ICL repair and holiday junction (HJ) resolution. Some of the phenotypes of FA patients could be attributed to a combination of defective ICL repair and HJ resolution, accounting for at least some of the broad range of symptoms ranging from pancytopenia to short stature and developmental delays [89].

### FANCA/XPF/Alpha II Spectrin interaction

Earlier work has shown that FANCA interacts with XPF and Alpha II Spectrin (aIISP) and that these three proteins co-localize to the nucleus in the case of ICL damage [90]. Because XPF has the ability to perform the dual incision step at the 5′ and 3′ locations flanking an ICL [91], it can be postulated that FANCA is at least partially responsible for coordinating and regulating this critical repair step in order to ensure ICL removal. This claim is further substantiated by the observation that FANCA^(−/−)^ cells are defective in this ICL dual incision step [92], suggesting that FANCA function is essential for the removal of these bulky lesions in order to maintain the integrity of the genetic code that they obstruct. It has been proposed that XPF–ERCC1 is the primary nuclease responsible for the unhooking step of ICL removal and that MUS81–EME1 plays a backup role in instances where XPF–ERCC1 is unable to perform its function. This has been speculated due to reduced sensitivity of MUS81–EME1 to crosslinking agents compared with XPF–ERCC1 deficient cells. MUS81–EME1 could also act during very specific instances of replication fork blockage that produce substrates for which it has preference, as in certain cases where the ICL is traversed and leading strand synthesis creates a 5′ flap on the 3′ side of an ICL [88]. Again, FANCA may serve as the regulatory component of these nuclease arrangements during ICL repair by determining which nuclease is required depending on the substrate present, and then subsequently recruiting or stimulating activity of the proper enzyme.

The potential significance of the interaction between FANCA and αIISP should not be ignored. αIISp is well known as a structural protein that associates with the nuclear matrix [93]. Previous work has suggested that the nuclear matrix may have a role in DNA damage repair, supported by the localization and assembly of NER factors to the nuclear matrix that is induced upon UV irradiation [94, 95]. Because XPF–ERCC1 is required for NER [96] and has also been shown to co-immunoprecipitate with FANCA and αIISp [90], it is likely that the repair activities facilitated by the nuclear matrix are important for genome maintenance in FA mediated pathways as well. It is proposed that αIISp acts as a scaffold to ensure proper assembly and alignment of ICL repair factors FANCA and XPF–ERCC1 during the incision step. Consistent with this, αIISp binds to DNA containing ICL damage and enhances the dual incision activity at these lesions. Additionally, FANCA, FANCB, FANCC, and FANCD2 deficient cells all exhibit lower αIISp levels, which results in reduced ICL repair compared with normal cells [97]. It appears that the relationship between FANCA and αIISp is important for increasing the efficiency of the ICL incision performed by XPF–ERCC1, perhaps through association with the nuclear matrix. It has been shown that FANCA and FANCC also form a complex with αIISp [98], yet the establishment of a role for the FA core or FA subcomplexes in the mechanism of αIISp related DDR (DNA damage response) remains to be defined. It has been discovered, however, that the regulation and stabilization of αIISp levels by FANCA [99] allows for another level of chromosomal maintenance. It has been shown that knockdown of αIISp levels to those present in FANCA deficient cells (35–40%) leads to a fivefold increase in chromosomal aberrations such as radials, breaks, and intrachromatid exchanges [100]. This indicates that regulation of αIISp by FA proteins is protective against chromosomal damage resulting from improperly processed ICL’s. Further research has revealed that the binding of FANCA and FANCG to the SH3 domain of αIISp prevents its degradation by μ-calpain, a protease that cleaves αIISp at Tyr1176 within repeat 11 [101, 102]. This inhibition is accomplished by blocking low-molecular-weight phosphotyrosine phosphatase (LMW-PTP) from dephosphorylating Tyr1176 and creating the available cleavage site for μ-calpain. FANCA and FANCG are also able to bind to μ-calpain, preventing its cleavage activity and allowing normal levels of αIISp to persist and carry out its functions in DNA repair. The loss of any of the FA proteins capable of blocking μ-calpain cleavage would then cause overactive breakdown of αIISp resulting in chromosomal instability. So far only FANCA and FANCG have been shown to physically interact with the SH3 domain of αIISp, but excess cleavage products of αIISp have been observed in FA-C, FA-D2, and FA-F cells so far [102]. The discovery of a DNA damage repair role for αIISp contributes to the elucidation of the full sequence of events that occur during resolution of ICL lesions. The proposed ability of αIISp to act as a scaffolding protein to promote incision activity also supports the individualized role of FANCA in mediating ICL removal along with XPF, although more work must be done in order to establish if, when, and how other FA proteins contribute to this process.

### FANCA/FEN1 interaction

FANCA has also been shown to stimulate the flap endonuclease activity of FEN1 with both 5′RNA flaps and DNA flaps as substrates [103]. FEN1 interacts with over 30 other proteins and is active in Okazaki fragment maturation, telomere maintenance, and replication fork rescue [104]. These functions and its aberrant expression in adenocarcinomas and other cancers have contributed to the general acceptance of FEN1 as a tumor suppressor gene. The interaction of FANCA with FEN1 could implicate a direct role in correct processing of Okazaki fragments. It is also possible that FANCA may work in concert with FEN1 in lagging strand synthesis through stabilization of the replication machinery while ensuring accurate copy of genetic information contained within Okazaki fragments. This is supported by co-localization of FANCA to replication forks in the absence of DNA damage [38, 103]. FANCA increases the efficiency of FEN1, possibly by loading it onto its substrate or competing for binding with its substrate, which could be responsible for increasing its turnover rate. It is possible that FANCA and FEN1 interact with each other in multiple processes due to the fact that FEN1 is stimulated by MUS81–EME1 in ICL unhooking and HJ resolution [105], two activities that FANCA has been proposed to participate in. Additionally, FANCA and FEN1 are both known to stabilize replication forks so it is likely that the two may work together in achieving this function.

### FANCA as a factor in resection-mediated repair pathways

FANCA has also shown itself to be an important factor for resection-mediated repair pathways. FANCA promotes homologous recombination as observed in a threefold reduction of GFP-positive FANCA null fibroblasts in an I-SceI based reporter assay that restores expression of GFP at a DSB site when repaired by HR [106]. FANCA could be supporting the homologous recombination route of repair through its interaction with BRCA1 via its N-terminal region [107], perhaps by recruiting, stabilizing or stimulating its activity as the role of this interaction is not clear in the context of DSB repair. It is not yet known whether promotion of HR involves other core complex proteins or not. In a similar assay, FANCA was also shown to be important in the single-stranded annealing pathway of repair (SSA) as seen by an approximate 50% decrease in SSA repair products at an I-SceI induced DSB in FANCA null fibroblasts [106]. This could be the result of FANCA’s role in a mechanism common to all modes of homology directed repair, or FANCA could specifically promote SSA under certain circumstances. The two main proteins known to mediate SSA are RAD52, which catalyzes the annealing step between homologous regions on resected ends at DSB; and RAD59 stimulates the annealing activity of RAD52 [107]. A direct interaction between FANCA and either of these two SSA proteins has yet to be shown, leaving much to be discovered about the actual activity carried out by FANCA in this repair pathway. Interestingly, studies have shown that XPF/ERCC1 functions as the flap endonuclease that removes the single-stranded non-homologous flaps generated from the formation of recombination intermediates during SSA [108, 109]. Because both FANCA and XPF/ERCC1 promote SSA and have been shown to co-localize in nuclear foci during ICL repair [90], perhaps the two carry out a comparable function when the SSA pathway takes place at a double-ended DSB. As mentioned previously, the ability of XPF to create incisions at an ICL lesion is defective in the absence of FANCA [92], indicating a stimulatory effect of FANCA on the nuclease activity of XPF. Therefore, it is feasible that FANCA interacts with XPF/ERCC1 in a similar manner during the flap removal step that follows annealing of homologous regions during SSA. Future studies will be required to discover exactly how FANCA participates in SSA and which proteins it interacts with in this repair process. More work also needs to be done to assess the conditions that regulate SSA activity because it is an error-prone pathway that must be tightly controlled in order to prevent dangerous genomic deletions.

It has also been recently discovered that FANCA participates in the alternative end-joining (Alt-EJ) method of DNA repair [110]. The previously referenced I-SceI/GFP reporter assay has shown that depletion of FANCA using SiRNA significantly decreased the amount of observed Alt-EJ in U2OS cells, while FANCA expression in mEF null cells increased the amount of repair product resulting from Alt-EJ [110]. This result may not have to do with individual FANCA activity itself, but rather the ability of the FA core complex to suppress NHEJ, which would allow Alt-EJ to occur. Support for this comes from the knockdown of other FANC proteins that displayed similar results as the FANCA knockdown. Although FANCA may promote Alt-EJ, Alt-EJ is not entirely dependent on FANCA because in FANCA null mEF (mouse embryonic fibroblast), Alt-EJ does still occur and is even increased by the further knockout of NHEJ factor Ku70 [110]. On the other hand, FANCA has shown the ability to stabilize regions of microhomology during Ig class switch recombination in B cells, which may translate to the ability of FANCA to recognize and stabilize duplexes throughout the genome during other processes mediated by microhomology such as Alt-EJ [111]. This could suggest a role for FANCA in promoting Alt-EJ without being entirely necessary for the pathway.

FANCA could also potentially be involved in the recruitment of other repair factors that promote the downstream steps of this pathway, such as the endonucleases that remove flap substrates resulting from heterologous tails that surround the homologous regions. An official flap-removal endonuclease has not yet been assigned to the Alt-EJ pathway. The XPF–ERCC1 homolog Rad1–Rad10 is able to cleave such heterologous tails in yeast, but the loss of XPF–ERCC1 does not cause a major decrease in Alt-EJ [112], which could mean that an additional protein is capable of carrying out this step. FANCA is able to regulate the catalytic activity of FEN1 [103] which has already been shown to contribute to Alt-EJ [113] and could feasibly act on the 5′ heterologous flaps resulting from the annealing step that are consistent with the structure-specific substrates on which FEN1 acts. Determining the factors that promote high-fidelity pathways of repair as opposed to error-prone mechanisms provide great insight into the conditions that permit the persistence of genome instability.

## Fanconi anemia proteins in mitigating oxidative stress

Reactive oxygen species (ROS) are a known source of DNA damage that can drive genomic instability. ROS such as hydroxyl radicals (OH^·^) can cause damage to all four nucleotide bases, and ^1^O_2_ can react with guanine producing carcinogenic alterations to DNA in the forms of mismatched bases, insertions, deletions, rearrangements, and chromosomal translocations characteristic of cancer-driving chromosomal instability [114]. 8-hydroxyguanine (8-OHG) or 8-oxo-2′-deoxyguanosine (8-oxo-dg) is the most commonly observed alteration resulting from ROS and the levels of these lesions are used to evaluate the amount of DNA damage occurring as a result of oxidative stress [114, 115]. Endogenous ROS are produced from the electron transport chain of mitochondria, lipid metabolism, and inflammatory cytokines while exogenous ROS can arise from ionizing radiation [116]. Damage from ROS occurring within a gene that is required for maintenance of genomic stability can effectively silence a tumor suppressor or other protein involved in DNA damage repair. ROS can also cause single or double-strand breaks of the DNA back bone, which can lead to loss of essential genetic information if not properly repaired [117]. An excess of DNA damage caused by ROS triggers p53 mediated apoptosis, and high levels of induced-cell death can lead to increased proliferation in order to replace the lost cells. This increased proliferation can provide a selective pressure for cells to evade apoptosis, which then results in genome instability and clonal selection of cells that harbor pro-oncogenic mutations [118].

### Evidence of FA proteins in regulating cellular oxidative stress

Disulphide linkage of FANCA and FANCG is induced concurrently with FANCD2 monoubiquitination in cells experiencing increased oxidative conditions, indicating a function for the FA pathway in responding to a harmful cellular environment caused by oxidative damage [119]. FA cells of differing complementation groups have also been shown to be hypersensitive to treatment with H_2_O_2_, a major source of ROS [119]. Signs of hypersensitivity range from elevated levels of 8-OHG in FANCC and FANCE deficient cell lines [120] to increased apoptosis in FANCA and FANCC deficient cells in pro-oxidant conditions [120, 121]. Although it may be true that FA proteins control oxidative DNA damage by participating in the repair of DNA lesions caused by ROS, there is also strong evidence that FA proteins are directly involved in regulating the amount of ROS and resulting oxidative DNA damage that persists within a cell. FA cells from groups A, C, and D2 display high levels of ROS and changes in mitochondria morphology that affect its roles in ATP synthesis and oxygen reuptake [122]. These misshapen mitochondria are then unable to produce ROS detoxifying enzymes such as Super Oxide Dismutase (SOD1), further allowing excess levels of ROS to accumulate [122]. Additionally, repair enzymes that function in the resolution of stalled replication forks can contribute to elevated levels of ROS that damage mitochondria, creating a vicious cycle of mitochondrial structural damage that results in unbridled ROS persistence [123]. The presence of excess ROS might also be a contributing factor to the cytoxicity of crosslinking agents in the case of FA deficiency. Support for this is shown by the ability for ROS scavengers, such as *N*-acetyl-1-cysteine (NAC), to ameliorate MMC sensitivity in FA cells [123]. Consistent with this claim, crosslinking agent DEB is able to induce oxidative DNA damage in the form of 8-OH-dG and the repair of DNA damage caused by DEB is dependent on antioxidant genes glutathione S-transferase (GST) and GSH peroxidase (GPx) [124]. Another source of ROS in FA cells stems from the overproduction of TNF-alpha and its direct effects on mitochondria, as well as its JNK-dependent ability to generate ROS through a positive feedback loop mechanism [125, 126]. The hypersensitivity of FANCC cells to TNF-alpha has been shown to cause increased apoptosis resulting in the clonal evolution that leads to AML. Restoration of FANCC expression protected cells from clonal evolution, while preventing excess ROS in these cells delayed leukemia development [127]. Sensitivity of overexpressed TNF-alpha and the increased ROS that it causes contributes to the genetic instability that leads to hematological malignancies in FA patients. The ability for ROS accumulation to exacerbate conditions already known to require FA protein intervention could at least partially explain the phenotypes observed in FA patients that are not present in diseases resulting from deficiencies in DNA repair proteins that function in similar pathways.

Multiple studies have confirmed biochemical activities of FA proteins in regulating the levels and damaging effects of ROS. The first evidence of direct FA protein capabilities in maintenance of cellular redox homeostasis came from the discovery of the interaction between FANCC and Cytochrome P450, a key enzyme in oxidative metabolism [128]. It was later found that FANCG interacts with cytochrome P4502E1 (CYP2E1), supporting direct roles for multiple FA proteins in redox metabolism [129]. Further research has found that H_2_O_2_ induces monoubiquitination of FANCD2, showing that the entire FA pathway is involved in an oxidative stress response, and also explaining the observed ROS sensitivity associated with mutations in complementation groups comprising the core complex [125].

### Protection of antioxidant gene promoters by the FA pathway

An interesting mechanism of FA proteins, specifically FANCA, in preventing cells from accumulation of ROS involves the protection of antioxidant gene promoters from oxidative stress [130]. DNA damage caused by ROS occurs selectively in promoter regions of several antioxidant genes such as *GCLC*, *TXNRD1*, *GSTP1* and *GPX1* in FA bone marrow (BM) cells, effectively down-regulating these protective cellular components, and contributing to the elevated levels of ROS observed in FA cells. 8-oxo-dG was the most common lesion observed, which is known to be highly mutagenic and capable of causing harmful transversions to genomic DNA. It was found that FANCA association with BRG1, the ATPase subunit of the BAF subcomplex in chromatin remodeling, greatly reduced the amount of oxidative damage to antioxidant promoters (*GPX1* and *TXNRD1*) compared with FA-A cells [130]. BRG1-FANCA mediated reduction in promoter oxidative damage was also dependent on monoubiquitinated FANCD2. In summary, FANCD2 activation of the FANCA-BRG1 complex is necessary for protection of oxidized bases in promoter regions of antioxidant genes through a type of chromatin remodeling activity [130].

### Ub-FANCD2 prevents TNF-alpha overexpression

FA cells are also deficient in neutralizing superoxide anions produced by elevated TNF-alpha levels [125]. The explanation for excess TNF-alpha levels in FA cells lies in the ability of the FA pathway to prevent NF-kB-mediated gene expression. The NF-kB transcription factor is able to up-regulate TNF-alpha levels through binding to the kB1 consensus site present in the TNF-alpha promoter region [131]. It has been shown that monoubiquitinated FANCD2 is able to functionally repress NF-kB transcriptional activity by binding to its kB1 consensus sequence within the distal site of the TNF-alpha promoter. The loss of inhibition of NF-kB induced gene expression allows unchecked TNF-alpha production that further generates harmful ROS. Activation of FANCD2 through monoubiquitination is required for its recruitment to the TNF-alpha promoter, but not for recognition of the NF-kB consensus site [125]. Additionally, FANCD2 deficiency allows for the overexpression of TNF-alpha that is observed in FA patients by allowing histone acetylation of the TNF-alpha promoter. The absence of FANCD2 results in increased apoptosis and high levels of DNA-damaging ROS [132]. The FANCD2 protein itself regulates ROS through a chromatin remodeling mechanism that allows for the deacetylation of histones within the TNF-alpha promoter in a monoubiquitination-independent manner [132]. The multiple roles of FA proteins in regulating the cellular oxidative state demonstrate the versatility of functions that they are able to utilize in order to protect the genome.

## Mitotic roles of Fanconi anemia proteins

Mitotic stress is a major contributor to genomic instability and cancer progression. The ability of cells to successfully segregate chromosomes and divide properly is equally essential to genomic integrity as proper genomic DNA replication. Aneuploidy is often present in solid tumors, and results from chromosome instability that usually stems from chromosome mis-segregation [133]. Mutated or aberrantly expressed proteins that participate in any of the tightly regulated steps conducting mitosis can cause chromosome instability. One of the features of Fanconi anemia cells across all disease mutations is the presence of aneuploidy and micronucleation, implicating a role for these proteins in ensuring faithful chromosome segregation.

### The FA/BLM relationship prevents aberrant chromosomal structures

One of the ways that the FA pathway prevents chromosome instability is by linking the recognition of replication stress to the resolution of chromosome abnormalities in mitosis through interaction with BLM [134]. Micronucleation occurs in FA cells during aphidicolin (APH) treatment, a drug that induces ultra-fine bridges (UFB) at common fragile sites (CFS), also known as difficult-to-replicate regions. Commonalities among the various CFSs have been difficult to decipher, but they are generally classified as ‘hot spots’ of genome instability where chromosome breakage and aberrant fusions frequently occur, and are often responsible for loss of tumor suppressors and oncogene amplifications [135, 136]. Earlier research has shown that cells with a disrupted FA pathway exhibit a two to threefold increase in chromosome breaks at known CFSs FRA3B and FRA16D, indicating the involvement of the FA pathway in maintaining the stability of these regions [137]. Functional FA pathway expression in fibroblasts has further been shown to rescue micronucleation caused by UFB at these CFSs, when compared with FA deficient fibroblasts [134]. The FA pathway has shown the ability to facilitate BLM repair function at anaphase bridges and faulty replication intermediates [134]. Anaphase bridges and UFBs are the structures that connect two daughter nuclei in replicating cells whose chromosomal DNA fails to separate, resulting in micronuclei and aneuploidy [138]. BLM has been shown to localize to these DNA-bridge structures and suppress their formation in normal cells [139]. The FA pathway has already demonstrated a common role with BLM in resolving replication stress, but there is also evidence to support that the FA/BLM relationship extends into mitotic genome maintenance as well. Confocal microscopy images have shown BLM bridges in normal cells connecting spots on segregating chromosomes where FANCD2 is located, and the amount of these BLM bridges increased upon APH or MMC treatment. Further analysis of the interaction between BLM and FANCD2 during mitosis revealed that BLM localization to non-centromeric anaphase bridges is compromised in FANC deficient cells, suggesting that the FA pathway is required for recruitment and/or stabilization of BLM at these APH-induced DNA structures [134] These capabilities indicate a role for the FA pathway in preventing mis-segregation of chromosomes when DNA lesions capable of compromising replication persist. It also further illustrates how FA proteins are involved in maintaining CFSs both independently and through collaboration with BLM [137]. While the FA pathway plays a substantial part in reducing UFB persistence, the exact roles played by FANCD2–FANCI foci and its functional interaction with BLM in this mechanism remain to be elucidated. Most recently, it has been reported that FANCD2 prevents CFS instability and facilitates replication through CFSs by ameliorating DNA:RNA hybrid accumulation and by influencing dormant origin firing [140].

### Proper regulation of the spindle assembly checkpoint by the FA pathway

The spindle assembly checkpoint (SAC) is responsible for coordinating proper destruction of sister chromatid cohesion and is able to halt the progression from metaphase to anaphase until appropriate kinetochore/microtubule attachment is ensured [133]. The FANC proteins co-localize to the mitotic apparatus during M phase and mutations in FA genes cause multinucleation in response to the chemotherapeutic agent taxol, a drug that functions as a spindle poison by stabilizing microtubules and disallowing them from attaching to kinetochores. The reintroduction of FANCA, specifically, is able to restore mitotic arrest and therefore SAC signaling in taxol-treated cells [141]. The FA proteins have also been shown to be partially responsible for maintaining correct centrosome numbers, confirmed by the presence of excess centrosomes upon pericentrin staining in primary patient-derived FA fibroblasts [141]. Abnormal centrosome number contributes to aneuploidy and chromosome instability by causing merotely during kinetochore/centrosome association, making centrosome maintenance important for genomic stability [133].

### Proper regulation of the SAC by FANCA

A more recent study confirmed that FANCA is crucial for regulating the SAC, and may play a more prominent role in this upkeep than the other FA proteins. FANCA null cells are able to escape the SAC and apoptosis upon treatment with taxol. In addition, FANCA proficient cells demonstrated increased cell cycle arrest and cell death upon taxol treatment [142]. This ability could suggest a mechanism by which an activated FANCA signaling pathway can prevent cancer in cells that do not satisfy the SAC by inducing apoptosis. Multinucleated cells were observed in FANCA KO cells upon treatment, indicating that a SAC compromised by loss of FANCA can cause chromosomal instability [142]. In the same study, FANCA demonstrated the ability to facilitate centrosome-mediated microtubule-spindle formation and growth. It was discovered that centrosomes in FANCA null fibroblasts emanated less microtubules with FANCA+ cells, showing that FANCA manages correct microtubule length in spindle assembly [142]. It will be interesting to explore if other FA proteins assist FANCA in these activities or if FANCA performs its mitotic roles independently.

## Mitotic protein interactions and roles of FANCA

### Centrosome number and NEK2

The cytoplasmic activity of FANCA reinforces its potential to carry out individual functions in mitosis [143]. FANCA also likely has a distinct role in centrosome maintenance, supported by its localization to the centrosome and its co-immunoprecipitation with gamma-tubulin. Further support of a centrosomal role for FANCA comes from the discovery of its phosphorylation by NEK2 at threonine-351 (T351) [144]. FANCA’s interaction with NEK2 is compelling due to the known ability of NEK2 in preserving centrosome integrity and its contributions to carcinogenesis. NEK2 is up-regulated in a variety of cancers such as breast cancer and lymphoma and has already been recognized as a potential therapeutic target for drug intervention [145]. More work must be done in order to establish the significance of the relationship between NEK2 and FANCA and the pathway in which they function, but this interaction does provide additional evidence to support centrosome maintenance activity for FANCA in centrosome maintenance. Consistent with this, FANCA T351 mutants display abnormal centrosome numbers, and are sensitive to the microtubule-interfering agent nocodazole. Correct centrosome number is important for ensuring faithful chromosome separation during cell division, which allows for genomic information to be properly passed down to daughter cells. In addition to sharing a common pathway with NEK2, siRNA knockdown of FANCA induces supernumerary centrosomes and mis-alignment of chromosomes during mitosis [144]. The evidence supporting FANCA regulation of centrosome number warrants further investigation into the mechanism of this function.

### Chromosome alignment and CENP-E

The N-terminus of FANCA directly interacts with the C-terminus of mitotic protein CENP-E [146]. CENP-E mediates microtubule/kinetochore attachments as well as chromosome congregation during mitosis [147]. CENP-E is important for ensuring proper chromosome segregation and correct chromosome numbers in daughter cells by acting as a motor protein to transport and align chromosomes at the spindle equator [148]. The exact role that FANCA plays with its binding partner CENP-E has not been determined, but exemplifies another potential area of interest involving FANCA’s regulation of mitotic processes to ensure chromosome fidelity in dividing cells. Improper chromosome congression can cause lagging chromosomes, a known phenotype of FANCA null cells [142]. Perhaps FANCA assists CENP-E in its assembly of chromosomes at the spindle equator, preventing the occurrence of improperly separated chromosomes.

### Potential mitotic FANCA/MUS81–EME1 function

It is possible that the regulation of FANCA on MUS81–EME1 has implications for maintaining genomic stability in early mitosis. MUS81–EME1 co-localizes to UFB resulting from common fragile sites along with FANCD2–FANCI in prometaphase, showing that MUS81–EME1 already works in concert with the FA pathway in this process. Depletion of MUS81 leads to an increased number of UFB stemming from CFS, highlighting its importance in maintaining chromosome fidelity at these CFSs prior to the completion of mitosis [149]. MUS81 has also been shown to induce programmed breaks at CFSs in late G2/early mitosis, a process that seems to be very important for successful sister chromatid separation [149]. Because FANCA has recently shown its ability to control the endonuclease activity of MUS81–EME1, it is feasible for FANCA to potentially regulate MUS81–EME1 in its cleavage activity at CFS in early mitosis. Creating programmed DNA breaks must be tightly regulated in order to prevent aberrant lesions, so other regulatory molecules most likely intervene in these processes in order to guarantee that these nucleases perform their cutting activity on the proper substrate at the appropriate time. FANCA has already been shown to regulate this activity of MUS81–EME1 at replication forks stalled by interstrand crosslinks [38]. FANCA has cytoplasmic activity with several demonstrated mitotic roles and the FA pathway has already shown the ability to maintain genomic CFS stability [137]. These characteristics support FANCA as a likely candidate to serve as a regulator of MUS81–EME1 incision activity at CFS during early mitosis. The multi-faceted capacities of FANCA support its relevance in providing genome stability in G2/M phase in addition to DNA replication during S phase. Apparently FANCA is more versatile than solely be part of the FA core complex that is involved in ICL or double strand break repair. We provide here a table as a brief summary of its known cellular functions discussed in this article (Table 1).

**Table 1:** Known cellular functions of FANCA

Pathway	Molecular action	Reference
DNA damage response		
Within the FA core complex	Part of A-G20 subcomplex, essential for the ubiquitination of FANCD2	[35]
	Intrinsically binds with ds and ssDNA, and RNA	[37]
	Phosphorylated at S1149, crucial for complex activity	[40]
	Involved in R-loop resolution	[35, 78]
	Promotes double strand break repair through homologous. Recombination and single strand annealing	[68, 106]
Out of the FA core complex	Regulates MUS81–EME1 incision activity at ICL	[38]
	Interacts with and regulates XPF’s incision activity at both 5′ and 3′ of ICL	[90, 92]
	SH3 mediated FANCA αIISP interaction stabilizes αIISP	[90, 101, 102]
	Promotes FEN1 endonuclease activity	[103]
Others		
Oxidative stress mitigation	Enhances cell survival in pro-oxidant conditions	[120, 121]
	Oxidative stress induced FANCA/BRG1/promoter complex protects antioxidant defense gene	[130]
Mitotic stress mitigation	Involved in the maintenance of normal spindle assembly	[142]
	T351 phosphorylation by NEK2 may plays a role in preserving centrosome integrity	[144]
	N terminus interacts with CENP-E and regulates chromosome alignment	[147]
Cell migration and motility	Modulates CXCR5 neddylation through an unknown mechanism and further stimulates cell migration and motility	[150]
	Direct and indirect transcriptional regulation through HES1, potential in promoting EMT	[151, 152]

## Conclusions and future directions

Understanding the DNA damage response’s impact on genome instability is crucial for advancing cancer research. There is a “malignant threshold” for the amount of assault the genome can handle before becoming at risk for oncogenic transformation [153]. Research has shown that the DNA damage response (DDR) (ATM-CHk2-p53) is over-active in pre-malignant tissues, and is also indicative of replicative stress [154]. This constitutive activation provides selective pressure for cells to acquire resistance to these checkpoints through a genetic instability mechanism conferred by such replication stress. Mutations in tumor suppressors or proto-oncogenes resulting from genome instability allow the evasion of apoptosis or senescence induced by the DDR, as previously mentioned in the instances of FA-driven AML. In order to maintain viability along with unrestrained growth and proliferation, cancer cells must walk a narrow path of allowing pro-oncogenic mutations while prohibiting a fatal amount of cytotoxicity. Because genomic instability seems to be necessary for this feat, understanding the molecular players that have a role in up-keeping this balance will be essential for determining the factors that allow malignant transformation to occur. Fanconi anemia proteins have functions in absolving the replication stress that promotes genomic instability, so greater knowledge of their involved pathways could provide helpful clues in elucidating the events that lead up to tumorigenesis.

The actions of FA proteins in protecting the genome could indicate their potential as therapeutic targets in drug discovery. Cancerous cells overcoming the DDR while preventing the threshold of damage that renders them unviable often leads to a dependence on certain DNA repair factors in the absence of others. The synthetic lethal approach in cancer drug development has become extremely popular due to this occurrence. Targeting the molecules for inhibition that cancer cells rely on to maintain a basal requirement of genomic stability has shown effectiveness in some specific cancers. The most popular example exploits the dependency of BRCA1 and BRCA2 deficient cancers on the base excision repair protein PARP1, leading to the development of PARP inhibitors (PARPi) [155]. PARPi have already made their way to clinical trials where they are showing promising results, especially in combination with other therapies such as chemotherapy, radiation, and CHK1 inhibitors [156]. The success of these personalized small molecule inhibitors has inspired researchers to search for the next therapeutic targets that specific cancers will be sensitive to, while having minimal effects on normal cells. It appears that the targets that seem to have the greatest potential are proteins that function in DNA damage repair, cell cycle regulation, and mitosis. Coincidentally, these are all pathways in which FA proteins also function. Previous attempts to develop Ku/DNA-PK inhibitors, ATR/CHK1 inhibitors, and Rad51 inhibitors have resulted in excessively cytotoxic and non-specific agents that are too impractical for clinical use [157]. Fanconi Anemia proteins have already demonstrated their potential to promote cancer growth and drug resistance in certain contexts. The dependence of BRCA1/2 cancers on FANCD2 in promoting Alt-EJ [74] makes exploitation of the FA pathway an attractive option for targeted therapies.

FANCA is able to promote error-prone repair pathways such as SSA that permit cancer-driving genomic instability. Manipulating this activity could be useful in preventing DNA damage repair in certain tumors that rely on these pathways, resulting in their death. Inhibiting the canonical FA pathway could have a myriad of toxic effects on cancer cells by sensitizing them to crosslinking agents or by inducing mitotic catastrophe through improper centrosome number regulation. Further research will be needed to evaluate the effects that targeting the FA pathway and its individual components will have on both cancerous cells as well as non-cancerous human tissues. In support of FA protein targeted therapy, it has been observed that the regulation of FA proteins does contribute to the success of tumors. Promoter hypermethylation of FANCF is observed in cases of AML [158] and ovarian cancer [159]. On the other hand, hypomethylation of FANCA promoters in squamous cell carcinoma of larynx (LSCC) cells has also been shown [160], which could mean that higher expression levels of these proteins contribute to oncogenic potential. Consistent with this, *FANCA* expression is up-regulated in basal breast tumors compared with non-basal breast tumors, and has higher expression levels in *RB1*-mutated retinoblastomas than *MYCN*-amplified retinoblastomas [161].

Studying FA proteins and the pathways in which they act might additionally explain some of the mechanisms used by cancer to alter cellular processes for their own benefit. The biochemical analysis of Fanconi anemia proteins has already provided a wealth of information detailing the many ways that cells preserve their sacred genetic code, but much more future research remains. Because altered levels of FA proteins have proven to be pathogenic, the study of how the activities of these proteins are regulated will assist in deciphering their full mechanisms of action. Exploring the genetic regulation and gene expression profiles of FA proteins could explain how their silencing or overexpression contributes to carcinogenesis. It has recently been discovered that p53 is able to down-regulate the FA pathway, and that high grade carcinomas (ovarian and adenocarcinomas) exhibit p53 loss and subsequent overexpression of at least 6FA proteins including FANCD2 and FANCA [162]. Whether this FA overexpression promotes cancerous pathways or not remains to be discovered but is nevertheless important for delineating the genetic changes that characterize tumor progression. Additional discoveries of epigenetic regulation, post-translational modifications, and regulatory binding partners will contribute to an understanding of how proper FA expression and activation protects the genome. There is a plethora of disease mutants to be studied that can expand further characterization of FA proteins’ biochemical properties. Protein, DNA, and RNA interactions that have already been discovered must be studied more in depth to establish significance in respective pathways. It has been over 20 years since the first FA protein was cloned [163], and a vast amount of information pertaining to their roles in hereditary disease as well as sporadic cancers through the enablement of genomic instability has been discovered through diligent research. Continuing to explore the functions of these proteins will provide more valuable insight into the cellular processes that protect our genome and govern our health, while also enlightening us to future therapeutic treatments for instability disorders and cancer.

## Abbreviations

FA: Fanconi anemia
MI: microsatellite instability
BER: base excision repair
NER: nucleotide excision repair
CIN: chromosomal instability
MMC: Mitomycin C
AML: acute myeloid leukemia
ICL: interstrand crosslink
NHEJ: nonhomologous end joining
SCE: sister chromatid exchange
MMEJ: microhomology mediated end joining
αIISP: Alpha II Spectrin
DDR: DNA damage response
SSA: single-stranded annealing
Alt-EJ: the alternative end-joining
ROS: reactive oxygen species
8-OHG: 8-hydroxyguanine
SAC: spindle assembly checkpoint

## References

[1] Pikor L, Thu K, Vucic E, Lam W (2013). The detection and implication of genome instability in cancer. Cancer Metastasis Rev.

[2] Negrini S, Gorgoulis VG, Halazonetis TD (2010). Genomic instability—an evolving hallmark of cancer. Nat Rev Mol Cell Biol.

[3] Halazonetis TD, Gorgoulis VG, Bartek J (2008). An oncogene-induced DNA damage model for cancer development. Science.

[4] Sawyer SL, Tian L, Kahkonen M, Schwartzentruber J, Kircher M, University of Washington Centre for Mendelian G (2015). Biallelic mutations in BRCA1 cause a new Fanconi anemia subtype. Cancer Discov..

[5] Dong H, Nebert DW, Bruford EA, Thompson DC, Joenje H, Vasiliou V (2015). Update of the human and mouse Fanconi anemia genes. Hum Genom..

[6] Bogliolo M, Surralles J (2015). Fanconi anemia: a model disease for studies on human genetics and advanced therapeutics. Curr Opin Genet Dev.

[7] Ceccaldi R, Sarangi P, D’Andrea AD (2016). The Fanconi anaemia pathway: new players and new functions. Nat Rev Mol Cell Biol.

[8] Bluteau D, Masliah-Planchon J, Clairmont C, Rousseau A, Ceccaldi R, Enghien DC (2016). Biallelic inactivation of REV7 is associated with Fanconi anemia. J Clin Investig..

[9] Kottemann MC, Smogorzewska A (2013). Fanconi anaemia and the repair of Watson and Crick DNA crosslinks. Nature.

[10] Pagano G, d’Ischia M, Pallardo FV (2015). Fanconi anemia (FA) and crosslinker sensitivity: re-appraising the origins of FA definition. Pediatr Blood Cancer.

[11] Kutler DI, Auerbach AD, Satagopan J, Giampietro PF, Batish SD, Huvos AG, Goberdhan A, Shah JP, Singh B (2003). High incidence of head and neck squamous cell carcinoma in patients with Fanconi anemia. Arch Otolaryngo Head Neck Surg..

[12] Alter BP, Greene MH, Velazquez I, Rosenberg PS (2003). Cancer in Fanconi anemia. Blood.

[13] Quentin S, Cuccuini W, Ceccaldi R, Nibourel O, Pondarre C, Pages MP (2011). Myelodysplasia and leukemia of Fanconi anemia are associated with a specific pattern of genomic abnormalities that includes cryptic RUNX1/AML1 lesions. Blood.

[14] Chen H, Zhang S, Wu Z (2014). Fanconi anemia pathway defects in inherited and sporadic cancers. Transl Pediatr..

[15] Smogorzewska A, Matsuoka S, Vinciguerra P, McDonald ER, Hurov KE, Luo J, Ballif BA, Gygi SP, Hofmann K, D’Andrea AD, Elledge SJ (2007). Identification of the FANCI protein, a monoubiquitinated FANCD2 paralog required for DNA repair. Cell.

[16] Alpi AF, Chaugule V, Walden H (2016). Mechanism and disease association of E2-conjugating enzymes: lessons from UBE2T and UBE2L3. Biochem J.

[17] Rickman KA, Lach FP, Abhyankar A, Donovan FX, Sanborn EM, Kennedy JA (2015). Deficiency of UBE2T, the E2 ubiquitin ligase necessary for FANCD2 and FANCI ubiquitination, causes FA-T subtype of Fanconi anemia. Cell Rep..

[18] Virts EL, Jankowska A, Mackay C, Glaas MF, Wiek C, Kelich SL (2015). AluY-mediated germline deletion, duplication and somatic stem cell reversion in UBE2T defines a new subtype of Fanconi anemia. Hum Mol Genet.

[19] Hira A, Yoshida K, Sato K, Okuno Y, Shiraishi Y, Chiba K (2015). Mutations in the gene encoding the E2 conjugating enzyme UBE2T cause Fanconi anemia. Am J Hum Genet.

[20] Lopez-Martinez D, Liang CC, Cohn MA (2016). Cellular response to DNA interstrand crosslinks: the Fanconi anemia pathway. Cell Mol Life Sci: CMLS..

[21] Boisvert RA, Howlett NG (2014). The Fanconi anemia ID2 complex: dueling saxes at the crossroads. Cell Cycle.

[22] Duxin JP, Walter JC (2015). What is the DNA repair defect underlying Fanconi anemia?. Curr Opin Cell Biol.

[23] Mamrak NE, Shimamura A, Howlett NG (2016). Recent discoveries in the molecular pathogenesis of the inherited bone marrow failure syndrome Fanconi anemia. Blood Rev.

[24] Park JY, Virts EL, Jankowska A, Wiek C, Othman M, Chakraborty SC, Vance GH, Alkuraya FS, Andreassen PR (2016). Complementation of hypersensitivity to DNA interstrand crosslinking agents demonstrates that XRCC2 is a Fanconi anaemia gene. J Med Genet.

[25] Cortez D (2015). Preventing replication fork collapse to maintain genome integrity. DNA Repair.

[26] Clauson C, Scharer OD, Niedernhofer L (2013). Advances in understanding the complex mechanisms of DNA interstrand cross-link repair. Cold Spring Harbor Perspect Biol..

[27] Sczepanski JT, Jacobs AC, Greenberg MM (2008). Self-promoted DNA interstrand cross-link formation by an abasic site. J Am Chem Soc.

[28] Semlow DR, Zhang J, Budzowska M, Drohat AC, Walter JC (2016). Replication-dependent unhooking of DNA interstrand cross-links by the NEIL3 glycosylase. Cell.

[29] Langevin F, Crossan GP, Rosado IV, Arends MJ, Patel KJ (2011). Fancd2 counteracts the toxic effects of naturally produced aldehydes in mice. Nature.

[30] Voulgaridou GP, Anestopoulos I, Franco R, Panayiotidis MI, Pappa A (2011). DNA damage induced by endogenous aldehydes: current state of knowledge. Mutat Res.

[31] Xie MZ, Shoulkamy MI, Salem AM, Oba S, Goda M, Nakano T, Ide H (2016). Aldehydes with high and low toxicities inactivate cells by damaging distinct cellular targets. Mutat Res.

[32] Zhang J, Dewar JM, Budzowska M, Motnenko A, Cohn MA, Walter JC (2015). DNA interstrand cross-link repair requires replication-fork convergence. Nat Struct Mol Biol.

[33] Long DT, Joukov V, Budzowska M, Walter JC (2014). BRCA1 promotes unloading of the CMG helicase from a stalled DNA replication fork. Mol Cell.

[34] Deans AJ, West SC (2011). DNA interstrand crosslink repair and cancer. Nat Rev Cancer.

[35] Huang Y, Leung JW, Lowery M, Matsushita N, Wang Y, Shen X, Huong D, Takata M, Chen J, Li L (2014). Modularized functions of the Fanconi anemia core complex. Cell Rep..

[36] Ali AM, Pradhan A, Singh TR, Du C, Li J, Wahengbam K, Grassman E, Auerbach AD, Pang Q, Meetei AR (2012). FAAP20: a novel ubiquitin-binding FA nuclear core-complex protein required for functional integrity of the FA-BRCA DNA repair pathway. Blood.

[37] Yuan F, Qian L, Zhao X, Liu JY, Song L, D’Urso G, Jain C, Zhang Y (2012). Fanconi anemia complementation group A (FANCA) protein has intrinsic affinity for nucleic acids with preference for single-stranded forms. J Biol Chem.

[38] Benitez A, Yuan F, Nakajima S, Wei L, Qian L, Myers R, Hu JJ, Lan L, Zhang Y (2014). Damage-dependent regulation of MUS81–EME1 by Fanconi anemia complementation group A protein. Nucleic Acids Res.

[39] Medhurst AL, Laghmani EH, Steltenpool J, Ferrer M, Fontaine C, de Groot J, Rooimans MA, Scheper RJ, Meetei AR, Wang W, Joenje H (2006). Evidence for subcomplexes in the Fanconi anemia pathway. Blood.

[40] Collins NB, Wilson JB, Bush T, Thomashevski A, Roberts KJ, Jones NJ, Kupfer GM (2009). ATR-dependent phosphorylation of FANCA on serine 1449 after DNA damage is important for FA pathway function. Blood.

[41] Bhagwat N, Olsen AL, Wang AT, Hanada K, Stuckert P, Kanaar R, D’Andrea A, Niedernhofer LJ, McHugh PJ (2009). XPF–ERCC1 participates in the Fanconi anemia pathway of cross-link repair. Mol Cell Biol.

[42] Bogliolo M, Schuster B, Stoepker C, Derkunt B, Su Y, Raams A (2013). Mutations in ERCC4, encoding the DNA-repair endonuclease XPF, cause Fanconi anemia. Am J Hum Genet.

[43] Hanada K, Budzowska M, Davies SL, van Drunen E, Onizawa H, Beverloo HB, Maas A, Essers J, Hickson ID, Kanaar R (2007). The structure-specific endonuclease Mus81 contributes to replication restart by generating double-strand DNA breaks. Nat Struct Mol Biol.

[44] Hanada K, Budzowska M, Modesti M, Maas A, Wyman C, Essers J, Kanaar R (2006). The structure-specific endonuclease Mus81–Eme1 promotes conversion of interstrand DNA crosslinks into double-strands breaks. EMBO J.

[45] Kim Y, Spitz GS, Veturi U, Lach FP, Auerbach AD, Smogorzewska A (2013). Regulation of multiple DNA repair pathways by the Fanconi anemia protein SLX4. Blood.

[46] Sengerova B, Wang AT, McHugh PJ (2011). Orchestrating the nucleases involved in DNA interstrand cross-link (ICL) repair. Cell Cycle.

[47] Wang AT, Sengerova B, Cattell E, Inagawa T, Hartley JM, Kiakos K (2011). Human SNM1A and XPF–ERCC1 collaborate to initiate DNA interstrand cross-link repair. Genes Dev.

[48] Castella M, Taniguchi T (2010). The role of FAN1 nuclease in the Fanconi anemia pathway. Cell Cycle.

[49] Liu T, Ghosal G, Yuan J, Chen J, Huang J (2010). FAN1 acts with FANCI–FANCD2 to promote DNA interstrand cross-link repair. Science.

[50] MacKay C, Declais AC, Lundin C, Agostinho A, Deans AJ, MacArtney TJ (2010). Identification of KIAA1018/FAN1, a DNA repair nuclease recruited to DNA damage by monoubiquitinated FANCD2. Cell.

[51] Shereda RD, Machida Y, Machida YJ (2010). Human KIAA1018/FAN1 localizes to stalled replication forks via its ubiquitin-binding domain. Cell Cycle.

[52] Smogorzewska A, Desetty R, Saito TT, Schlabach M, Lach FP, Sowa ME, Clark AB, Kunkel TA, Harper JW, Colaiácovo MP, Elledge SJ (2010). A genetic screen identifies FAN1, a Fanconi anemia-associated nuclease necessary for DNA interstrand crosslink repair. Mol Cell.

[53] Yoshikiyo K, Kratz K, Hirota K, Nishihara K, Takata M, Kurumizaka H, Horimoto S, Takeda S, Jiricny J (2010). KIAA1018/FAN1 nuclease protects cells against genomic instability induced by interstrand cross-linking agents. Proc Natl Acad Sci USA.

[54] Hodskinson MR, Silhan J, Crossan GP, Garaycoechea JI, Mukherjee S, Johnson CM, Schärer OD, Patel KJ (2014). Mouse SLX4 is a tumor suppressor that stimulates the activity of the nuclease XPF–ERCC1 in DNA crosslink repair. Mol Cell.

[55] Budzowska M, Graham TG, Sobeck A, Waga S, Walter JC (2015). Regulation of the Rev1–pol zeta complex during bypass of a DNA interstrand cross-link. EMBO J.

[56] Raschle M, Knipscheer P, Enoiu M, Angelov T, Sun J, Griffith JD, Ellenberger TE, Schärer OD, Walter JC (2008). Mechanism of replication-coupled DNA interstrand crosslink repair. Cell.

[57] Newell AE, Akkari YM, Torimaru Y, Rosenthal A, Reifsteck CA, Cox B, Grompe M, Olson SB (2004). Interstrand crosslink-induced radials form between non-homologous chromosomes, but are absent in sex chromosomes. DNA Repair (Amst)..

[58] Adamo A, Collis SJ, Adelman CA, Silva N, Horejsi Z, Ward JD, Martinez-Perez E, Boulton SJ, La Volpe A (2010). Preventing nonhomologous end joining suppresses DNA repair defects of Fanconi anemia. Mol Cell.

[59] Renaud E, Barascu A, Rosselli F (2016). Impaired TIP60-mediated H4K16 acetylation accounts for the aberrant chromatin accumulation of 53BP1 and RAP80 in Fanconi anemia pathway-deficient cells. Nucleic Acids Res.

[60] Murina O, von Aesch C, Karakus U, Ferretti LP, Bolck HA, Hanggi K, Sartori AA (2014). FANCD2 and CtIP cooperate to repair DNA interstrand crosslinks. Cell Rep..

[61] Wang LC, Stone S, Hoatlin ME, Gautier J (2008). Fanconi anemia proteins stabilize replication forks. DNA Repair (Amst)..

[62] Sobeck A, Stone S, Costanzo V, de Graaf B, Reuter T, de Winter J (2006). Fanconi anemia proteins are required to prevent accumulation of replication-associated DNA double-strand breaks. Mol Cell Biol.

[63] Lachaud C, Moreno A, Marchesi F, Toth R, Blow JJ, Rouse J (2016). Ubiquitinated Fancd2 recruits Fan1 to stalled replication forks to prevent genome instability. Science.

[64] Kratz K, Schopf B, Kaden S, Sendoel A, Eberhard R, Lademann C, Cannavó E, Sartori AA, Hengartner MO, Jiricny J (2010). Deficiency of FANCD2-associated nuclease KIAA1018/FAN1 sensitizes cells to interstrand crosslinking agents. Cell.

[65] Zhou W, Otto EA, Cluckey A, Airik R, Hurd TW, Chaki M (2012). FAN1 mutations cause karyomegalic interstitial nephritis, linking chronic kidney failure to defective DNA damage repair. Nat Genet.

[66] Hickson ID (2003). RecQ helicases: caretakers of the genome. Nat Rev Cancer.

[67] Meetei AR, Sechi S, Wallisch M, Yang D, Young MK, Joenje H, Hoatlin ME, Wang W (2003). A multiprotein nuclear complex connects Fanconi anemia and Bloom syndrome. Mol Cell Biol.

[68] Deans AJ, West SC (2009). FANCM connects the genome instability disorders Bloom’s syndrome and fanconi anemia. Mol Cell.

[69] Panneerselvam J, Wang H, Zhang J, Che R, Yu H, Fei P (2016). BLM promotes the activation of Fanconi anemia signaling pathway. Oncotarget..

[70] Hoadley KA, Xue Y, Ling C, Takata M, Wang W, Keck JL (2012). Defining the molecular interface that connects the Fanconi anemia protein FANCM to the Bloom syndrome dissolvasome. Proc Natl Acad Sci USA.

[71] Michl J, Zimmer J, Tarsounas M (2016). Interplay between Fanconi anemia and homologous recombination pathways in genome integrity. EMBO J.

[72] Iliakis G, Murmann T, Soni A (2015). Alternative end-joining repair pathways are the ultimate backup for abrogated classical non-homologous end-joining and homologous recombination repair: implications for the formation of chromosome translocations. Mutat Res, Genet Toxicol Environ Mutagen.

[73] Verma P, Greenberg RA (2016). Noncanonical views of homology-directed DNA repair. Genes Dev.

[74] Kais Z, Rondinelli B, Holmes A, O’Leary C, Kozono D, D’Andrea AD, Ceccaldi R (2016). FANCD2 maintains fork stability in BRCA1/2-deficient tumors and promotes alternative end-joining DNA repair. Cell Rep..

[75] Skourti-Stathaki K, Proudfoot NJ (2014). A double-edged sword: R loops as threats to genome integrity and powerful regulators of gene expression. Genes Dev.

[76] Aguilera A, Garcia-Muse T (2012). R loops: from transcription byproducts to threats to genome stability. Mol Cell.

[77] Skourti-Stathaki K, Kamieniarz-Gdula K, Proudfoot NJ (2014). R-loops induce repressive chromatin marks over mammalian gene terminators. Nature.

[78] Garcia-Rubio ML, Perez-Calero C, Barroso SI, Tumini E, Herrera-Moyano E, Rosado IV, Aguilera A (2015). The Fanconi anemia pathway protects genome integrity from R-loops. PLoS Genet.

[79] Schwab RA, Nieminuszczy J, Shah F, Langton J, Lopez Martinez D, Liang CC, Cohn MA, Gibbons RJ, Deans AJ, Niedzwiedz W (2015). The Fanconi anemia pathway maintains genome stability by coordinating replication and transcription. Mol Cell.

[80] Auerbach AD (2009). Fanconi anemia and its diagnosis. Mutat Res.

[81] Moldovan GL, D’Andrea AD (2009). How the fanconi anemia pathway guards the genome. Annu Rev Genet.

[82] Vaz F, Hanenberg H, Schuster B, Barker K, Wiek C, Erven V (2010). Mutation of the RAD51C gene in a Fanconi anemia-like disorder. Nat Genet.

[83] Wang AT, Smogorzewska A (2015). SnapShot: Fanconi anemia and associated proteins. Cell.

[84] Faivre L, Guardiola P, Lewis C, Dokal I, Ebell W, Zatterale A (2000). Association of complementation group and mutation type with clinical outcome in fanconi anemia. European Fanconi Anemia Research Group. Blood.

[85] Adachi D, Oda T, Yagasaki H, Nakasato K, Taniguchi T, D’Andrea AD, Asano S, Yamashita T (2002). Heterogeneous activation of the Fanconi anemia pathway by patient-derived FANCA mutants. Hum Mol Genet.

[86] Lo Ten Foe JR, Rooimans MA, Bosnoyan-Collins L, Alon N, Wijker M, Parker L (1996). Expression cloning of a cDNA for the major Fanconi anaemia gene, FAA. Nat Genet.

[87] Garcia-Higuera I, Kuang Y, Denham J, D’Andrea AD (2000). The fanconi anemia proteins FANCA and FANCG stabilize each other and promote the nuclear accumulation of the Fanconi anemia complex. Blood.

[88] Zhang J, Walter JC (2014). Mechanism and regulation of incisions during DNA interstrand cross-link repair. DNA Repair.

[89] Larin M, Gallo D, Tamblyn L, Yang J, Liao H, Sabat N, Brown GW, McPherson JP (2014). Fanconi anemia signaling and Mus81 cooperate to safeguard development and crosslink repair. Nucleic Acids Res.

[90] Sridharan D, Brown M, Lambert WC, McMahon LW, Lambert MW (2003). Nonerythroid alphaII spectrin is required for recruitment of FANCA and XPF to nuclear foci induced by DNA interstrand cross-links. J Cell Sci.

[91] Fisher LA, Bessho M, Bessho T (2008). Processing of a psoralen DNA interstrand cross-link by XPF–ERCC1 complex in vitro. J Biol Chem.

[92] Kumaresan KR, Lambert MW (2000). Fanconi anemia, complementation group A, cells are defective in ability to produce incisions at sites of psoralen interstrand cross-links. Carcinogenesis.

[93] Bachs O, Lanini L, Serratosa J, Coll MJ, Bastos R, Aligue R, Rius E, Carafoli E (1990). Calmodulin-binding proteins in the nuclei of quiescent and proliferatively activated rat liver cells. J Biol Chem.

[94] Koehler DR, Hanawalt PC (1996). Recruitment of damaged DNA to the nuclear matrix in hamster cells following ultraviolet irradiation. Nucleic Acids Res.

[95] Balajee AS, May A, Bohr VA (1998). Fine structural analysis of DNA repair in mammalian cells. Mutat Res.

[96] Su Y, Orelli B, Madireddy A, Niedernhofer LJ, Scharer OD (2012). Multiple DNA binding domains mediate the function of the ERCC1–XPF protein in nucleotide excision repair. J Biol Chem.

[97] McMahon LW, Sangerman J, Goodman SR, Kumaresan K, Lambert MW (2001). Human alpha spectrin II and the FANCA, FANCC, and FANCG proteins bind to DNA containing psoralen interstrand cross-links. Biochemistry.

[98] McMahon LW, Walsh CE, Lambert MW (1999). Human alpha spectrin II and the Fanconi anemia proteins FANCA and FANCC interact to form a nuclear complex. J Biol Chem.

[99] Brois DW, McMahon LW, Ramos NI, Anglin LM, Walsh CE, Lambert MW (1999). A deficiency in a 230 kDa DNA repair protein in fanconi anemia complementation group A cells is corrected by the FANCA cDNA. Carcinogenesis.

[100] McMahon LW, Zhang P, Sridharan DM, Lefferts JA, Lambert MW (2009). Knockdown of alphaII spectrin in normal human cells by siRNA leads to chromosomal instability and decreased DNA interstrand cross-link repair. Biochem Biophys Res Commun..

[101] Lefferts JA, Wang C, Sridharan D, Baralt M, Lambert MW (2009). The SH3 domain of alphaII spectrin is a target for the Fanconi anemia protein, FANCG. Biochemistry.

[102] Zhang P, Sridharan D, Lambert MW (2010). Knockdown of mu-calpain in Fanconi anemia, FA-A, cells by siRNA restores alphaII spectrin levels and corrects chromosomal instability and defective DNA interstrand cross-link repair. Biochemistry.

[103] Qian L, Yuan F, Rodriguez-Tello P, Padgaonkar S, Zhang Y (2013). Human Fanconi anemia complementation group a protein stimulates the 5′ flap endonuclease activity of FEN1. PLoS ONE.

[104] Zheng L, Jia J, Finger LD, Guo Z, Zer C, Shen B (2011). Functional regulation of FEN1 nuclease and its link to cancer. Nucleic Acids Res.

[105] Shin YK, Amangyeld T, Nguyen TA, Munashingha PR, Seo YS (2012). Human MUS81 complexes stimulate flap endonuclease 1. FEBS J.

[106] Yang YG, Herceg Z, Nakanishi K, Demuth I, Piccoli C, Michelon J, Hildebrand G, Jasin M, Digweed M, Wang ZQ (2005). The Fanconi anemia group A protein modulates homologous repair of DNA double-strand breaks in mammalian cells. Carcinogenesis.

[107] Folias A, Matkovic M, Bruun D, Reid S, Hejna J, Grompe M, D’Andrea A, Moses R (2002). BRCA1 interacts directly with the Fanconi anemia protein FANCA. Hum Mol Genet.

[108] Adair GM, Rolig RL, Moore-Faver D, Zabelshansky M, Wilson JH, Nairn RS (2000). Role of ERCC1 in removal of long non-homologous tails during targeted homologous recombination. EMBO J.

[109] Al-Minawi AZ, Saleh-Gohari N, Helleday T (2008). The ERCC1/XPF endonuclease is required for efficient single-strand annealing and gene conversion in mammalian cells. Nucleic Acids Res.

[110] Howard SM, Yanez DA, Stark JM (2015). DNA damage response factors from diverse pathways, including DNA crosslink repair, mediate alternative end joining. PLoS Genet.

[111] Nguyen TV, Riou L, Aoufouchi S, Rosselli F (2014). Fanca deficiency reduces A/T transitions in somatic hypermutation and alters class switch recombination junctions in mouse B cells. J Exp Med.

[112] Bennardo N, Cheng A, Huang N, Stark JM (2008). Alternative-NHEJ is a mechanistically distinct pathway of mammalian chromosome break repair. PLoS Genet.

[113] Liang L, Deng L, Chen Y, Li GC, Shao C, Tischfield JA (2005). Modulation of DNA end joining by nuclear proteins. J Biol Chem.

[114] Wiseman H, Halliwell B (1996). Damage to DNA by reactive oxygen and nitrogen species: role in inflammatory disease and progression to cancer. Biochem J.

[115] Zhu X, Hondroulis E, Liu W, Li CZ (2013). Biosensing approaches for rapid genotoxicity and cytotoxicity assays upon nanomaterial exposure. Small.

[116] Valko M, Rhodes CJ, Moncol J, Izakovic M, Mazur M (2006). Free radicals, metals and antioxidants in oxidative stress-induced cancer. Chemico-Biol Interact..

[117] Cooke MS, Evans MD, Dizdaroglu M, Lunec J (2003). Oxidative DNA damage: mechanisms, mutation, and disease. FASEB J.

[118] Lensch MW, Tischkowitz M, Christianson TA, Reifsteck CA, Speckhart SA, Jakobs PM (2003). Acquired FANCA dysfunction and cytogenetic instability in adult acute myelogenous leukemia. Blood.

[119] Park SJ, Ciccone SL, Beck BD, Hwang B, Freie B, Clapp DW, Lee SH (2004). Oxidative stress/damage induces multimerization and interaction of Fanconi anemia proteins. J Biol Chem.

[120] Zunino A, Degan P, Vigo T, Abbondandolo A (2001). Hydrogen peroxide: effects on DNA, chromosomes, cell cycle and apoptosis induction in Fanconi’s anemia cell lines. Mutagenesis.

[121] Saadatzadeh MR, Bijangi-Vishehsaraei K, Hong P, Bergmann H, Haneline LS (2004). Oxidant hypersensitivity of Fanconi anemia type C-deficient cells is dependent on a redox-regulated apoptotic pathway. J Biol Chem.

[122] Kumari U, Ya Jun W, Huat Bay B, Lyakhovich A (2014). Evidence of mitochondrial dysfunction and impaired ROS detoxifying machinery in Fanconi anemia cells. Oncogene.

[123] Lyakhovich A (2013). Damaged mitochondria and overproduction of ROS in Fanconi anemia cells. Rare Dis..

[124] Pagano G, Talamanca AA, Castello G, Pallardo FV, Zatterale A, Degan P (2012). Oxidative stress in Fanconi anaemia: from cells and molecules towards prospects in clinical management. Biol Chem..

[125] Du W, Erden O, Pang Q (2014). TNF-alpha signaling in Fanconi anemia. Blood Cells Mol Dis.

[126] Ventura JJ, Cogswell P, Flavell RA, Baldwin AS, Davis RJ (2004). JNK potentiates TNF-stimulated necrosis by increasing the production of cytotoxic reactive oxygen species. Genes Dev.

[127] Li J, Sejas DP, Zhang X, Qiu Y, Nattamai KJ, Rani R, Rathbun KR, Geiger H, Williams DA, Bagby GC, Pang Q (2007). TNF-alpha induces leukemic clonal evolution ex vivo in Fanconi anemia group C murine stem cells. J Clin Invest..

[128] Kruyt FA, Hoshino T, Liu JM, Joseph P, Jaiswal AK, Youssoufian H (1998). Abnormal microsomal detoxification implicated in Fanconi anemia group C by interaction of the FAC protein with NADPH cytochrome P450 reductase. Blood.

[129] Futaki M, Igarashi T, Watanabe S, Kajigaya S, Tatsuguchi A, Wang J, Liu JM (2002). The FANCG Fanconi anemia protein interacts with CYP2E1: possible role in protection against oxidative DNA damage. Carcinogenesis.

[130] Du W, Rani R, Sipple J, Schick J, Myers KC, Mehta P, Andreassen PR, Davies SM, Pang Q (2012). The FA pathway counteracts oxidative stress through selective protection of antioxidant defense gene promoters. Blood.

[131] Collart MA, Baeuerle P, Vassalli P (1990). Regulation of tumor necrosis factor alpha transcription in macrophages: involvement of four kappa B-like motifs and of constitutive and inducible forms of NF-kappa B. Mol Cell Biol.

[132] Matsushita N, Endo Y, Sato K, Kurumizaka H, Yamashita T, Takata M, Yanagi S (2011). Direct inhibition of TNF-alpha promoter activity by Fanconi anemia protein FANCD2. PLoS ONE.

[133] Thompson SL, Bakhoum SF, Compton DA (2010). Mechanisms of chromosomal instability. Curr Biol: CB..

[134] Naim V, Rosselli F (2009). The FANC pathway and BLM collaborate during mitosis to prevent micro-nucleation and chromosome abnormalities. Nat Cell Biol.

[135] Arlt MF, Casper AM, Glover TW (2003). Common fragile sites. Cytogenet Gen Res..

[136] Ozeri-Galai E, Bester AC, Kerem B (2012). The complex basis underlying common fragile site instability in cancer. Trends Genet: TIG..

[137] Howlett NG, Taniguchi T, Durkin SG, D’Andrea AD, Glover TW (2005). The Fanconi anemia pathway is required for the DNA replication stress response and for the regulation of common fragile site stability. Hum Mol Genet.

[138] Gisselsson D, Pettersson L, Hoglund M, Heidenblad M, Gorunova L, Wiegant J, Mertens F, Dal Cin P, Mitelman F, Mandahl N (2000). Chromosomal breakage-fusion-bridge events cause genetic intratumor heterogeneity. Proce Natl Acad Sci USA.

[139] Chan KL, North PS, Hickson ID (2007). BLM is required for faithful chromosome segregation and its localization defines a class of ultrafine anaphase bridges. EMBO J.

[140] Madireddy A, Kosiyatrakul ST, Boisvert RA, Herrera-Moyano E, Garcia-Rubio ML, Gerhardt J (2016). FANCD2 facilitates replication through common fragile sites. Mol Cell.

[141] Nalepa G, Enzor R, Sun Z, Marchal C, Park SJ, Yang Y, Tedeschi L, Kelich S, Hanenberg H, Clapp DW (2013). Fanconi anemia signaling network regulates the spindle assembly checkpoint. J Clin Invest..

[142] Abdul-Sater Z, Cerabona D, Potchanant ES, Sun Z, Enzor R, He Y, Robertson K, Goebel WS, Nalepa G (2015). FANCA safeguards interphase and mitosis during hematopoiesis in vivo. Exp Hematol.

[143] Ferrer M, Rodriguez JA, Spierings EA, de Winter JP, Giaccone G, Kruyt FA (2005). Identification of multiple nuclear export sequences in Fanconi anemia group A protein that contribute to CRM1-dependent nuclear export. Hum Mol Genet.

[144] Kim S, Hwang SK, Lee M, Kwak H, Son K, Yang J, Kim SH, Lee CH (2013). Fanconi anemia complementation group A (FANCA) localizes to centrosomes and functions in the maintenance of centrosome integrity. Int J Biochem Cell Biol.

[145] Frett B, Brown RV, Ma M, Hu W, Han H, Li HY (2014). Therapeutic melting pot of never in mitosis gene a related kinase 2 (Nek2): a perspective on Nek2 as an oncology target and recent advancements in Nek2 small molecule inhibition. J Med Chem.

[146] Du J, Chen L, Shen J (2009). Identification of FANCA as a protein interacting with centromere-associated protein E. Acta Biochim Biophys Sin.

[147] Gudimchuk N, Vitre B, Kim Y, Kiyatkin A, Cleveland DW, Ataullakhanov FI, Grishchuk EL (2013). Kinetochore kinesin CENP-E is a processive bi-directional tracker of dynamic microtubule tips. Nat Cell Biol.

[148] Foley EA, Kapoor TM (2009). Chromosome congression: on the bi-orient express. Nat Cell Biol.

[149] Ying S, Minocherhomji S, Chan KL, Palmai-Pallag T, Chu WK, Wass T, Mankouri HW, Liu Y, Hickson ID (2013). MUS81 promotes common fragile site expression. Nat Cell Biol.

[150] Renaudin X, Guervilly JH, Aoufouchi S, Rosselli F (2014). Proteomic analysis reveals a FANCA-modulated neddylation pathway involved in CXCR5 membrane targeting and cell mobility. J Cell Sci.

[151] Tremblay CS, Huard CC, Huang FF, Habi O, Bourdages V, Levesque G, Carreau M (2009). The fanconi anemia core complex acts as a transcriptional co-regulator in hairy enhancer of split 1 signaling. J Biol Chem.

[152] Wang SC, Lin XL, Wang HY, Qin YJ, Chen L, Li J (2015). Hes1 triggers epithelial-mesenchymal transition (EMT)-like cellular marker alterations and promotes invasion and metastasis of nasopharyngeal carcinoma by activating the PTEN/AKT pathway. Oncotarget..

[153] Khanna A (2015). DNA damage in cancer therapeutics: a boon or a curse?. Cancer Res.

[154] Bartkova J, Horejsi Z, Koed K, Kramer A, Tort F, Zieger K (2005). DNA damage response as a candidate anti-cancer barrier in early human tumorigenesis. Nature.

[155] Chen A (2011). PARP inhibitors: its role in treatment of cancer. Chin J Cancer..

[156] Booth L, Cruickshanks N, Ridder T, Dai Y, Grant S, Dent P (2013). PARP and CHK inhibitors interact to cause DNA damage and cell death in mammary carcinoma cells. Cancer Biol Ther.

[157] Gavande NS, VanderVere-Carozza PS, Hinshaw HD, Jalal SI, Sears CR, Pawelczak KS, Turchi JJ (2016). DNA repair targeted therapy: the past or future of cancer treatment?. Pharmacol Ther.

[158] Tischkowitz M, Ameziane N, Waisfisz Q, De Winter JP, Harris R, Taniguchi T, D’Andrea A, Hodgson SV, Mathew CG, Joenje H (2003). Bi-allelic silencing of the Fanconi anaemia gene FANCF in acute myeloid leukaemia. Br J Haematol.

[159] Olopade OI, Wei M (2003). FANCF methylation contributes to chemoselectivity in ovarian cancer. Cancer Cell.

[160] Szaumkessel M, Richter J, Giefing M, Jarmuz M, Kiwerska K, Tonnies H, Grenman R, Heidemann S, Szyfter K, Siebert R (2011). Pyrosequencing-based DNA methylation profiling of Fanconi anemia/BRCA pathway genes in laryngeal squamous cell carcinoma. Int J Oncol.

[161] Haitjema A, Mol BM, Kooi IE, Massink MP, Jorgensen JA, Rockx DA, Rooimans MA, de Winter JP, Meijers-Heijboer H, Joenje H, Dorsman JC (2014). Coregulation of FANCA and BRCA1 in human cells. SpringerPlus..

[162] Jaber S, Toufektchan E, Lejour V, Bardot B, Toledo F (2016). p53 downregulates the Fanconi anaemia DNA repair pathway. Nat Commun..

[163] Strathdee CA, Gavish H, Shannon WR, Buchwald M (1992). Cloning of cDNAs for Fanconi’s anaemia by functional complementation. Nature.

[164] Lightfoot J, Alon N, Bosnoyan-Collins L, Buchwald M (1999). Characterization of regions functional in the nuclear localization of the Fanconi anemia group A protein. Hum Mol Genet.

